# Blood pressure in primary school children in Uganda: a cross-sectional survey

**DOI:** 10.1186/1471-2458-14-1223

**Published:** 2014-11-26

**Authors:** Farah Kidy, Diana Rutebarika, Swaib A Lule, Moses Kizza, Amos Odiit, Emily L Webb, Alison M Elliott

**Affiliations:** London School of Hygiene and Tropical Medicine, Keppel Street, London, WC1E 7HT UK; MRC/UVRI Uganda Research Unit on AIDS, P.O. Box 49, Entebbe, Uganda; Department of Paediatrics & Child Health, Makerere University, College of Health Sciences, P.O BOX 7072, Kampala, Uganda; The Surgery @ Aylestone, 672 Aylestone Road, Leicester, LE2 8PR UK; St Mary’s Family Clinic, P.O.Box 23610, Kampala, Uganda; Department of Paediatrics & Child Health; Faculty of Medicine, University of Rwanda, P.O. BOX 117, Butare, Rwanda

**Keywords:** Blood pressure, Hypertension, Children, Uganda, Africa

## Abstract

**Background:**

Non-communicable diseases are an emerging concern in sub-Saharan Africa, and risks for these conditions are often based on exposures in early life, with premonitory signs developing during childhood. The prevalence of hypertension has been reported to be high in African adults, but little is known about blood pressure in African children. We studied prevalence and risk factors for high blood pressure (HBP) among school children in central Uganda.

**Methods:**

Two urban and five rural schools were randomly selected from government schools in Wakiso district, Uganda. Questionnaires were administered and anthropometric measures taken. Blood pressure (BP) was measured three times in one sitting (on day 1) and the average compared to internationally-used normograms. Children with BP >95th percentile were re-tested at two additional sittings (day 2 and day 3) within one week, and at two further follow up visits over a period of six months. Those with sustained HBP were referred for further investigation.

**Results:**

Of 552 students included, 539 completed the initial assessments (days 1–3) of whom 92 (17.1%) had HBP at the initial sitting. Age (adjusted odds ratio (aOR) 1.29 (95% confidence interval 1.14, 1.47), p< 0.001), body mass index (1.70 (1.25-2.31) p = 0.001) and soil-transmitted helminths (2.52 (1.04-6.11), 0.04) were associated with increased prevalence of HBP at the initial sitting. After further investigation, sustained HBP was seen in 14 children, yielding an estimated prevalence of 3.8% allowing for losses to follow up. Four children required treatment.

**Conclusion:**

It is feasible to measure blood pressure accurately in the school setting. A high HBP prevalence on initial readings gave cause for concern, but follow up suggested a true HBP prevalence commensurate with international normograms. Extended follow up is important for accurate assessment of blood pressure among African children.

## Background

There is growing recognition of the importance of non‒communicable diseases (NCDs) in developing countries [1]. The World Health Organisation (WHO) Global Burden of Disease report states that 25% of all deaths in Africa are due to NCDs, with cardiovascular conditions contributing nearly half those deaths [2]. It is estimated that 77.1 million women and 73.6 million men in sub-Saharan Africa will be hypertensive by 2025 [3]. Sustained elevated blood pressure (BP) is a major risk factor for stroke, coronary heart disease and renal disease. Post‒mortem studies have shown that high blood pressure (HBP) in childhood, especially high systolic BP, is a risk factor for the development of fatty streaks and fibrous plaques in the coronary arteries of young people [4]. Longitudinal studies following children from age seven to young adulthood have demonstrated that high systolic and diastolic readings in childhood are predictive of hypertension in adulthood [5].

There have been limited attempts to estimate the prevalence of childhood HBP in Africa [6, 7]. A recent review of HBP demonstrated prevalences ranging from 7.5% to 22.3% in data largely from South Africa [6]. Although secondary HBP (resulting from pathological insults) constitutes a higher proportion of HBP cases in children than in adults, primary HBP is still commoner than secondary HBP in children in developed countries, with obesity playing a major role [8]. The relative contribution of primary and secondary HBP in children in developing countries is unknown.

This school-based, cross-sectional survey, conducted in Uganda, addressed the procedures required to assess blood pressure accurately in primary school children in this setting, and investigated risk factors for high blood pressure associated with rural or urban environments: parasitic infections, body mass index (BMI) and other risk factors.

## Methods

This cross-sectional study was conducted in Entebbe Municipality and Ssisa sub-country, Wakiso district, Uganda in 2010. In Uganda, in 2010 [9], school enrolment in our target age group was estimated at 84%, with 4.4 million boys and 4.3 million girls enrolled. Entebbe Municipality is a relatively urbanised community close to Uganda’s international airport and the capital, Kampala. The socioeconomic background of the inhabitants ranges from wealthy commuters to poor fishing communities. Ssisa sub-county comprises predominantly rural communities.

Schools in Entebbe Municipality (purposively selected to represent a relatively urban community) and in Sissa subcounty (purposively selected to represent a rural community) provided the sampling frame for the study. Only schools receiving government support were included in the sampling frame, armed forces schools and one school catering exclusively for special needs children were excluded. Schools were then selected at random from the resulting list of eligible schools. Two urban schools were selected from a total of nine eligible schools, and five rural schools, also from a total of nine eligible schools. In selected schools, all children in primary classes three, four and five were eligible for inclusion (except in one rural school, where, on the advice of the head teacher, children in primary classes two, three and four were included as likely to be more similar in age to the other schools). These classes were chosen as children were thought to be old enough to answer questionnaires and participate in the activities of the project, but avoided disruption to the highest classes, preparing for national examinations. No exclusion criteria were applied to children within the classes.

Ethical approval was obtained from the Science and Ethics Committee of the Uganda Virus Research Institute, the Uganda National Council for Science and Technology and the London School of Hygiene and Tropical Medicine. Informed written consent was obtained from parents of all participants in English or Luganda. Children also provided assent.

In the urban schools, ten boys and ten girls from each class were randomly selected to be tested for malaria and helminth infections by taking a systematic sample from the list of children for whom consent and assent had been obtained. In the smaller rural schools all participants were tested.

Data and samples were collected at an initial set of visits on three separate days (“day 1”, “day 2”, “day 3”; all within one week) in July or September 2010 and then at two subsequent single day follow-up visits at intervals of approximately three months (“follow up 1” and “follow up 2”).

The team received training for a period of one week in the use of the equipment, issues pertaining to questionnaire delivery and confidentiality. During the initial set of visits, age, gender and tympanic temperature were recorded. Height was measured to the nearest millimetre using a measuring pole. Weight was measured to the nearest 0.1 kilogram using electronic scales, without shoes or heavy clothing. Body mass index (BMI – weight/height^2^), BMI-for-age percentile and z-score, and height-for-age percentile and z-score were calculated using WHO Anthroplus version 1.0.3. Most children were not aware of their date of birth; these children were designated to be their reported age plus 6 months.

A questionnaire, designed to obtain simple information on the child’s health, and risk factors for HBP, was administered to all participants in English or Luganda.

Blood pressure was measured on the right arm using an Omron M6 automated upper arm blood pressure monitor with appropriate cuff size for the child’s arm [10]. Oscillometric devices were used because of their ease of use and also to minimise inter-observer variability and digit preference. Although no local validation of Omron M6 was done, a palpation method of blood pressure determination was used in addition for the few children sent to Mulago hospital before recommendations were made upon management.

Participants sat for five minutes before BP measurements were commenced. Three readings were taken at intervals of at least five minutes. For each child, the mean of the three systolic BPs and the mean of the three diastolic BPs were calculated. These readings were compared to internationally-used normograms [11] on the basis of age, gender and height percentile. If the mean systolic or diastolic measurement was ≥95th percentile for a child on the day 1 visit, then the process was repeated both day 2 and day 3, within one week. HBP was defined as having average mean systolic and/or diastolic readings, taken on all three different days, that were ≥95th percentile. If the mean systolic and diastolic BPs taken on day 1 were normal (below the 95^th^ percentile) then no further readings were taken.

Children diagnosed with HBP during these initial visits were seen at two subsequent follow-up visits (“follow up 1” and “follow up 2”) at intervals of approximately three months. BP readings were repeated three times in one sitting at each follow-up visit. Parents were encouraged to attend the second follow-up visit. Children with mean systolic or diastolic BP ≥95^th^ percentile on the three initial visits and at both follow up visits were defined as having sustained HBP and were investigated to identify underlying causes using urine analysis by dipstick, full blood count, renal function tests, chest x-ray and renal ultra sound. Referrals were made to AO at Mulago National Referral Hospital with transport provided.

Two thick blood films were taken from the selected children and prepared on the same slide. The slides were read independently by two trained lab assistants. Stool samples were collected from the same children. Two Kato‒Katz slides were prepared from each sample and examined within 30 minutes for hookworm eggs and at a later stage for ova of other species. All children with malaria or helminth infections received treatment and their parents were informed in writing.

We aimed to invite 360 children in rural and 360 children in urban schools to enrol in the study. No preliminary data were available to allow power calculations for the HBP outcome. Data entry and analysis were carried out using Epi‒Info version 6 and Stata version 12. The prevalence of HBP based on data from the initial visits was calculated overall, and by each risk factor category. Differences between distribution of risk factors in urban and rural schools were examined using odds ratios and chi-squared tests. Crude associations between each risk factor [age, gender, BMI z-score, family history of high blood pressure, cigarette smoke exposure, rural/urban environment, malaria and helminth infections (analysed by subtype as *Schistosoma mansoni* or soil-transmitted helminths)] and the outcome of HBP were assessed using logistic regression to calculate odds ratios, 95% confidence intervals and p-values. Multivariable analysis was then conducted using logistic regression, adjusting for risk factors that were crudely associated with the outcome of interest at p< 0.2, and that were not considered to be on the causal pathway between the risk factor of interest and the outcome. Associations between each risk factor and mean systolic blood pressure on “day 1” and between each risk factor and mean diastolic blood pressure on “day 1” were also investigated, using simple and multiple linear regression, and a similar approach to multivariable analysis as described above.

## Results

Five rural and two urban schools were selected. A total of 381 and 611 children were on the register in selected classes in the rural and urban schools, respectively. The proportion of those registered who enrolled in the study varied between schools (24% to 73% in rural schools, 48% to 71% in urban schools). Lack of enrolment was mainly due to children being absent on the days the study was conducted and to inaccuracies in the class registers. Five hundred and fifty‒two students were enrolled (184 from rural and 368 from urban schools, Table 1). They ranged in age from 7 to 18 years with a mean age of 11 years. Two hundred and ninety-six of the participants were female (53.6%). Children in rural schools, compared to their urban counterparts, were more likely to have malaria (odds ratio (OR) 3.83 (95% confidence interval 2.08-7.03) p< 0.001) and to be exposed to cigarette smoke (1.54 (1.01-2.36) p = 0.05), but less likely to be infected with schistosomes (0.24 (0.08-0.70) p = 0.01) and less likely to be overweight or obese (p = 0.03).

**Table 1:** **Characteristics of study participants**

Variable	Category	Rural schools (n = 184)	Urban schools (n = 368)	P-value ^a^
Age	7-8 years	10 (5.4%)	23 (6.3%)	0.58
	9-10 years	56 (30.4%)	129 (35.1%)	
	11-12 years	71 (38.6%)	125 (34.0%)	
	13-14 years	43 (23.4%)	77 (20.9%)	
	15-18 years	4 (2.2%)	14 (3.8%)	
Gender	Male	94 (51.1%)	162 (44.0%)	0.12
	Female	90 (48.9%)	206 (56.0%)	
BMI-for-age Z-score^b^	Underweight	4 (2.2%)	6 (1.6%)	0.03
	Normal	163 (90.1%)	298 (81.6%)	
	Overweight	14 (7.7%)	57 (15.6%)	
	Obese	0 (0%)	4 (1.1%)	
Family history of high blood pressure	None	98 (53.3%)	222 (60.3%)	0.28
	Any	67 (36.4%)	112 (30.4%)	
	Not known	19 (10.3%)	34 (9.2%)	
Cigarette smoke exposure	Unexposed	134 (74.0%)	295 (81.5%)	0.05
	Exposed	47 (26.0%)	67 (18.5%)	
Malaria^c^	Uninfected	111 (62.7%)	103 (86.6%)	<0.001
	Infected	66 (37.3%)	16 (13.5%)	
*Schistosoma mansoni* ^c^	Uninfected	171 (97.2%)	99 (89.2%)	0.01
	Infected	5 (2.8%)	12 (10.8%)	
Soil transmitted helminths^c^	Uninfected	158 (89.8%)	98 (89.1%)	0.86
	Infected	18 (10.2%)	12 (10.9%)	

^a^P-value for difference in distribution of characteristic between children from rural and urban schools; ^b^Categorisation of BMI-for-age Z-score using WHO-recommended thresholds: underweight (Z-score< -2), normal (-2 ≤ Z-score<1), overweight (1 ≤ Z-score<2), obese (Z-score ≥2); ^c^Malaria, *Schistosoma mansoni* and soil transmitted helminth infection status were ascertained for a randomly selected sample of 20 children per class in the urban schools; Missing values in rural and urban school participants, respectively: BMI-for-age Z-score 3, 3; cigarette smoke exposure 3, 6; malaria 7, 1; *Schistosoma mansoni* 8, 9; soil transmitted helminths 8, 10.

**Figure 1:**
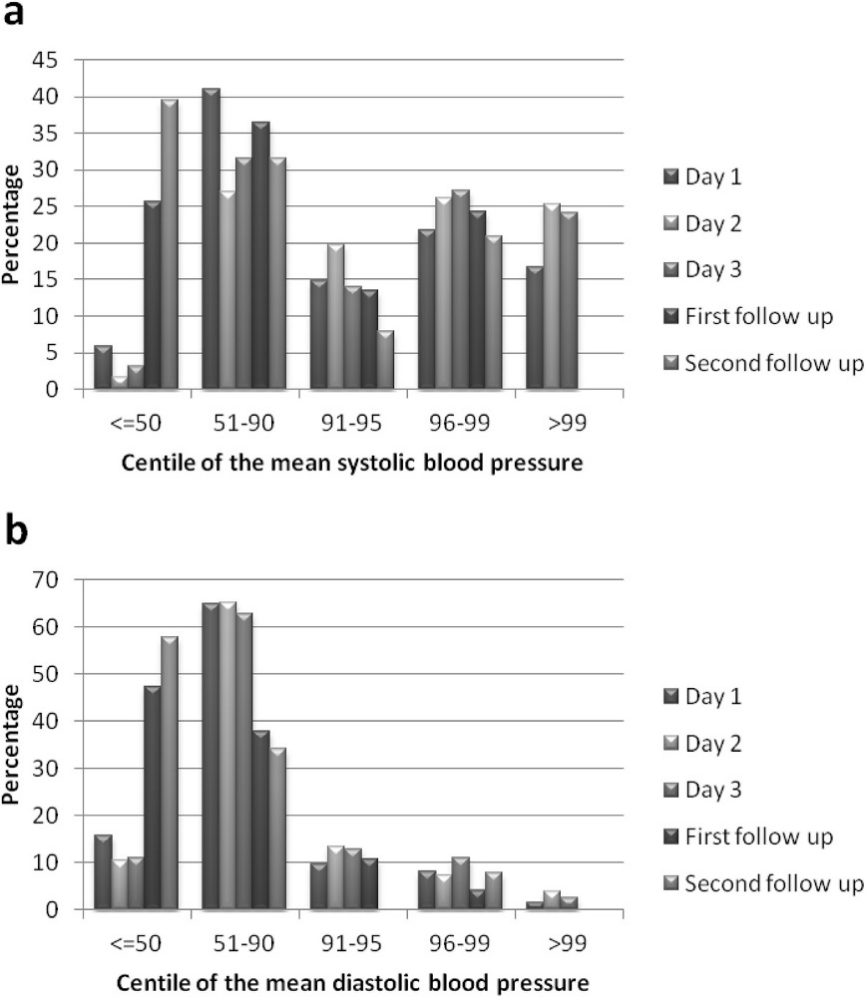
**Distribution of normogram-based centiles of the mean systolic (a) and mean diastolic (b) blood pressure readings taken during the initial and follow up visits.** The number of children examined at each visit is shown in Figure 2.

The mean (standard deviation; SD) systolic BP on day 1 was 116.9 mmHg (12.4 mmHg). The mean (SD) diastolic BP was 68.0 mmHg (8.7 mmHg). On day 1, 94.5% of the mean systolic results and 84.2% of the mean diastolic results were above the 50^th^ centile based on internationally used normograms. A similar distribution was seen when children with day 1 BP readings above the 95 percentile were re-measured on subsequent days in the same week (day 2 and day 3; Figure 1). However when children diagnosed with HBP based on days 1, 2 and 3 were seen again at follow up after approximately three and then six months, the distribution of pressures had become more normal: at the final visit, about half of the children seen had pressures below the fiftieth centile, as expected from a normal distribution (Figure 1).

Of the 539 children for whom data were available from the initial visits (days 1–3), 92 (17.1%, 95% CI: 13.9% to 20.2%) were found to have HBP. Increasing age (adjusted (a)OR 1.29(1.14-1.47), p< 0.001), increasing BMI Z-score (aOR 1.70 (1.25-2.31), p = 0.001) and soil transmitted helminth infection (aOR 2.52 (1.04-6.11), p = 0.04) were associated with an increased prevalence of HBP based on the initial visits (Table 2). Results analysing mean systolic blood pressure and mean diastolic blood pressure on day 1 as outcomes were similar (Table 3), with increasing age and BMI both strongly positively associated with systolic and diastolic blood pressure. However soil transmitted helminth infection was not associated with either systolic or diastolic blood pressure (p = 0.48 and 0.49 respectively). On the other hand, children infected with malaria had on average lower systolic and diastolic blood pressure than uninfected children. Girls had somewhat higher mean systolic blood pressure than boys, while children from urban schools had reduced mean diastolic blood pressure compared to children from rural schools.

**Figure 2:**
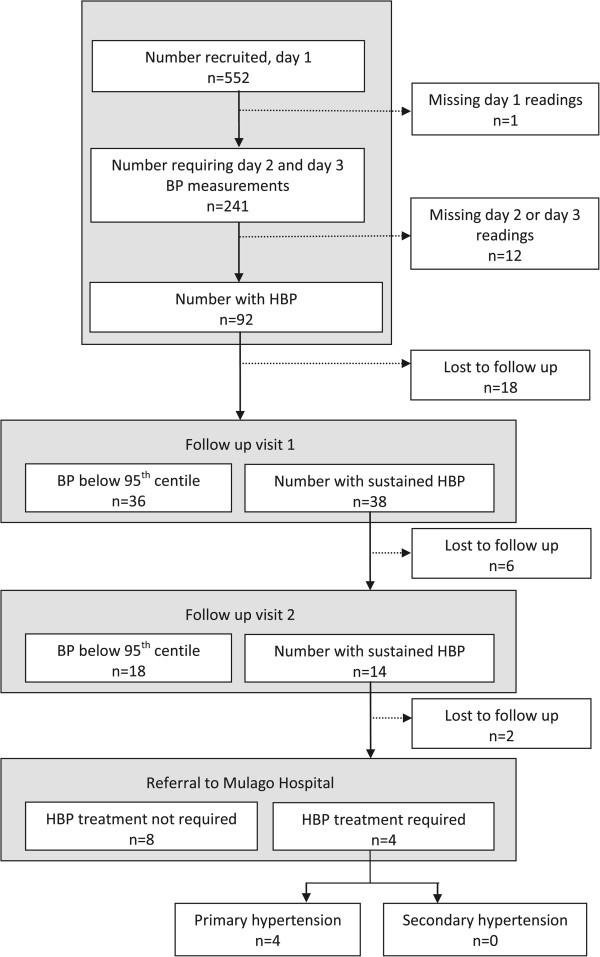
**The flow of participants through the blood pressure protocol procedures.**

The flow of participants through the study is seen in Figure 2. Of the 92 children with HBP based on the initial set of visits, 74 were present when the schools were visited for follow up visit 1 and 38/74 (51.3%) still had HBP. Thirty-two of these 38 children were seen on follow up visit 2. Mean systolic and/or diastolic BP remained elevated in 14/32 (43.8%), thus sustained HBP was confirmed in 2.6% (14/539; 95% CI: 1.2% to 4%) of the original participants. Among the 14 participants, three and eight had diastolic blood pressure above the 95th and 99th percentile, respectively, and three had systolic blood pressure above the 99th percentile. Allowing for losses to follow up and assuming the proportions at each stage of the protocol are representative, we estimate the prevalence of sustained HBP to be 3.8% (95% CI: 2.4%-5.9%).

**Table 2:** **Factors associated with high blood pressure among school children in Wakiso District, Uganda**

Variable		High blood pressure, n (%)	Crude OR (95% CI)	P	Adjusted OR (95% CI) ^b^	P
Age			1.31 (1.16, 1.49)	<0.001	1.29 (1.14, 1.47)	<0.001
Gender	Male	35/249 (14.1%)	1		1	
	Female	57/290 (19.7%)	1.50 (0.94,2.37)	0.09	1.37 (0.85, 2.21)	0.20
BMI Z score			1.82 (1.34, 2.47)^c^	<0.001	1.70 (1.25, 2.31)^c^	0.001
Family history of high blood pressure	None	52/312 (16.7%)	1	0.47	-	
	Any	34/177 (19.2%)	1.19 (0.74, 1.92)			
	Not known	6/50 (12.0%)	0.68 (0.28, 1.68)			
Cigarette smoke exposure	Unexposed	75/419 (17.9%)	1		-	
	Exposed	17/114 (14.9%)	0.80 (0.45, 1.43)	0.45		
Rural or urban school	Rural	30/179 (16.8%)	1		-	
	Urban	62/360 (17.2%)	1.03 (0.64, 1.67)	0.89		
Malaria^a^	Uninfected	44/207 (21.3%)	1		1	
	Infected	10/82 (12.2%)	0.51 (0.25, 1.08)	0.08	0.63 (0.28, 1.43)	0.27
*Schistosoma mansoni* ^a^	Uninfected	52/263 (19.8%)	1		-	
	Infected	3/17 (117.6%)	0.87 (0.24, 3.14)	0.83		
Soil transmitted helminths^a^	Uninfected	45/249 (18.1%)	1		1	
	Infected	10/30 (33.3%)	2.27 (0.99, 5.17)	0.05	2.52 (1.04, 6.11)	0.04

^a^Malaria, *schistosoma mansoni* and soil transmitted helminth infection status were ascertained for a randomly selected sample of 20 children per class in the urban schools; ^b^Odds ratios for age, gender and BMI were adjusted for each other but not for malaria and soil transmitted helminths; odds ratios for malaria and soil transmitted helminths were adjusted for age, gender, BMI and each other; ^c^BMI Z score was analysed as a continuous covariate.

**Table 3:** **Factors associated with systolic and diastolic blood pressure among school children in Wakiso District, Uganda**

Variable		Mean (SD)	Mean difference (95% CI)	P	Adjusted mean difference (95% CI) ^b^	P
***Systolic blood pressure***						
Age			3.24 (2.73, 3.75)	<0.001	3.14 (2.63, 3.65)	<0.001
Gender	Male	115.9 (12.5)	reference		reference	
	Female	117.7 (12.3)	1.79 (-0.28, 3.87)	0.09	1.81 (0.01, 3.61)	0.05
BMI Z score			3.29 (2.14, 4.43)^c^	<0.001	2.78 (1.74, 3.83)^c^	<0.001
Family history of high blood pressure	None	116.5 (12.7)	reference	0.13	reference	0.97
	Any	118.0 (11.9)	1.49 (-0.76, 3.75)		0.25 (-1.71, 2.21)	
	Not known	114.2 (11.2)	-2.34 (-6.01, 1.34)		-0.01 (-3.19, 3.18)	
Cigarette smoke exposure	Unexposed	117.4 (12.2)	reference		reference	
	Exposed	114.7 (12.6)	-2.68 (-5.22, -0.13)	0.04	-1.03 (-3.25, 1.18)	0.36
Rural or urban school	Rural	116.8 (10.8)	reference			
	Urban	117.0 (13.1)	0.22 (-1.98, 2.42)	0.84		
Malaria^a^	Uninfected	119.1 (12.0)	reference		reference	
	Infected	113.7 (1.06)	-5.40 (-8.37, -2.43)	<0.001	-3.79 (-6.31, -1.27)	0.003
*Schistosoma mansoni* ^a^	Uninfected	117.8 (11.8)	reference			
	Infected	115.8 (13.3)	-2.05 (-7.91, 3.80)	0.49		
Soil transmitted helminths^a^	Uninfected	117.4 (12.0)	reference			
	Infected	120.0 (10.6)	2.52 (-2.00, 7.04)	0.27		
***Diastolic blood pressure***						
Age			1.53 (1.14, 1.91)	<0.001	1.48 (1.10, 1.85)	<0.001
Gender	Male	67.6 (8.9)	reference		reference	
	Female	68.3 (8.5)	0.73 (-0.74, 2.19)	0.33	0.86 (-0.50, 2.23)	0.22
BMI Z score			1.56 (0.73, 2.38)	<0.001	1.64 (0.85, 2.43)	<0.001
Family history of high blood pressure	None	67.5 (9.2)	reference	0.20		
	Any	68.8 (8.1)	1.39 (-0.20, 2.98)			
	Not known	67.3 (7.3)	-0.14 (-2.74, 2.45)			
Cigarette smoke exposure	Unexposed	68.0 (8.5)	reference			
	Exposed	67.6 (9.1)	-0.39 (-2.18, 1.40)	0.67		
Rural or urban school	Rural	69.8 (7.8)	reference		reference	
	Urban	67.1 (9.0)	-2.73 (-4.26, -1.20)	<0.001	-3.08 (-4.54, -1.62)	<0.001
Malaria^a^	Uninfected	69.8 (8.7)	reference		reference	
	Infected	66.9 (8.2)	-2.93 (-5.11, -0.74)	0.009	-2.84 (-4.97, -0.72)	0.009
*Schistosoma mansoni* ^a^	Uninfected	69.3 (8.6)	reference			
	Infected	67.8 (9.7)	-1.52 (-5.78, 2.75)	0.48		
Soil transmitted helminths^a^	Uninfected	69.1 (8.8)	reference			
	Infected	70.9 (6.9)	1.80 (-1.49, 5.09)	0.28		

^a^Malaria, *Schistosoma mansoni* and soil transmitted helminths infection status were ascertained for a randomly selected sample of 20 children per class in the urban schools; ^b^For associations with systolic blood pressure, mean differences for age, gender, BMI and cigarette smoke exposure were adjusted for each other but not for malaria; mean difference for malaria was adjusted for age, gender, BMI and cigarette smoke exposure. For associations with diastolic blood pressure, mean differences for age, gender, BMI and rural/urban school were adjusted for each other but not for malaria; mean difference for malaria was adjusted for age, gender, BMI and rural/urban school; ^c^BMI Z score was analysed as a continuous covariate.

Children with sustained HBP were invited for review by the paediatricians at Mulago Hospital. Four of the twelve who were reviewed were considered to require treatment. Among children with persistently high blood pressure, urine dipstick, full blood count and creatinine results were normal for all tested. The chest x-ray was abnormal, showing cardiomegaly, for one child. We were unable to identify a secondary cause of hypertension in any of the children with sustained HBP.

Six children who did not meet our criteria for HBP but who had a strong family history of HBP and readings over the 90^th^ centile were also investigated at the referral centre, on the advice of the consultant (AO) (not included in the flow diagram; Figure 2). In one of these children, renal ultrasound demonstrated one kidney smaller than age-appropriate norms. This child subsequently required treatment for HBP.

## Discussion

In this survey among primary school children in Uganda, the initial prevalence of HBP, assessed by measurement in triplicate on three separate days within one week, was high at 17.1%. This is comparable with recent results from adults in Uganda, all from studies in which blood pressure was assessed by two or three repeated measurements on a single day (14.6% to 22%) [12–15]. However, only a minority of our children had sustained HBP on extended follow up. Our estimated prevalence for sustained HBP was 3.8% (95% CI: 2.4%-5.9%). This is in keeping with the figure of 5% expected to have blood pressure readings above the 95% percentile. This suggests that, while population-specific normograms might be preferable, the international normograms used may be reasonably applicable in this setting.

The initial negative skew in our data, which resolved on extended follow up, emphasises the importance of repeated measurements over a considerable time period to diagnose HBP in children. Blood pressure varies with circadian rhythms and in response to activity or emotion. These fluctuations are more marked in children (especially around puberty) than in adults [11]. As a result, in order to obtain an accurate picture of a child’s blood pressure, it is recommended that repeated measurements are taken at several different sittings on different days [16]. As far as we are aware, ours is the first survey among children in sub-Saharan Africa to have included such extended follow up. Studies from South Africa have determined BP in children using a protocol similar to our day 1 procedure, with repeated measurements on a single day, and demonstrated HBP prevalence varying between 7.5% and 22.3% [6], comparable to our initial estimate of 17.1%. Data from North America collected over a longer period, suggest a prevalence of HBP between 2% and 5% [11] comparable to our estimate of sustained HBP of 3.8%.

The initial negative skew also implies that care is needed in interpreting the implications of the associations between risk factors and BP results obtained during the initial three-day testing (in our study) and at a single sitting (in other studies). However, subject to this caveat, in keeping with our results, a study in Zambia found that increasing age was associated with higher blood pressure amongst rural adolescents [17]. We found a weak association with female gender, also, but this was only statistically significant for systolic blood pressure. A limitation of our study was that the stage of puberty was not assessed. Studies from Ghana [18] and Nigeria [19] found that increasing body mass index and urban locality were independently associated with higher blood pressure, but a diagnosis of hypertension was not made. We also designed our study to make rural–urban comparisons, and found some evidence that urban locality was associated with lower diastolic blood pressure. This finding was not replicated for the systolic blood pressure or HBP outcomes, thus it may be a chance finding. Our urban population was sampled from Entebbe and although the children attended urban schools, their levels of poverty meant that, for some, their home lives closely resembled a rural existence (the relatively high prevalence of schistosomiasis in Entebbe children emphasises this). Our rural sample was smaller than intended due to a variety of factors including smaller schools and poor attendance.

We found that malaria infection was associated with lower blood pressure on day 1, although this did not translate to an association with HBP based on the full set of initial visits. To our knowledge, no other study has reported such an association, and this may simply be a reflection of the well known association between clinical malaria and low blood pressure, reflected to a lesser degree in these asymptomatic children. Our finding that soil transmitted helminth infections were associated with increased HBP is surprising (although analysing systolic and diastolic blood pressure on day 1 found no evidence of an association).

Some of these findings may be due to residual confounding: we were unable to account for socioeconomic status in our analysis, and this may be important since a study in Kinshasa, in the Democratic Republic of Congo, showed that elevated blood pressure was associated with low socio‒economic status (OR = 1.2; 95% CI 1.1 to 1.3; p<0.01) [7]. Poverty is generally associated with poor hygiene and increased risk of soil‒transmitted helminths and malaria [20]. In future, larger studies among African children would be of interest to determine whether factors associated with HBP on a short-term testing protocol are similar to those associated with sustained HBP, but our sample size was too small for this.

It is interesting to note that most cases of sustained HBP were likely to be primary HBP, even in this population with little obesity and generally high levels of physical activity. This may be the result of genetics or of nutritional factors such as high salt consumption that were not determined in this study: further investigation is needed. Although it can be difficult to assess heart size using x-rays in children, the report of cardiomegaly on chest x-ray in one case suggests that HBP may have been present for a sufficient period, and at sufficient intensity, to result in cardiac strain.

Identification of children with HBP in this setting was a relatively easy step. However, provision of follow up and treatment presents a challenge. For us, investigation proved difficult to arrange despite the fact that it was offered free by the research programme. None of the parents of the four children identified as requiring treatment anticipated that they would be able to afford the prescribed drugs.

According to Ministry of Education data, only 68.4% of eligible children in Wakiso district were enrolled in primary schools at the time of this study [21], and out-of-school children were not sampled. At the opposite extreme, children at private schools were not sampled and these may, at present, be the group most at risk from NCD risk factors associated with changing lifestyles. These two groups need to be considered in future studies.

## Conclusion

There is a lack of information about the prevalence of HBP and of the risk factors leading to HBP in tropical sub-Saharan Africa. While studies in adults suggest a high prevalence of hypertension, this study among children, with extended follow up, suggested a prevalence of HBP in keeping with international norms. However, a less meticulous study, confined to a short-term, three-day protocol for BP assessment, would have resulted in a major over-estimate of HBP in this population. School-based surveys for NCD risks are feasible and informative in this setting, but rigorous protocols need to be followed for the assessment of HBP in children.
